# Indents

**Published:** 1905-05-15

**Authors:** 


### INDENTS.

**Brief Articles of Technical or Scientific Value will receive notice and comment. Contributions invited.**

**Separating Medium.** Dissolve paraffin in gasoline to saturation. Apply to impression lightly with a camel's hair brush.—*Dental Hints.*

**Phenol-Sulphonic Acid.** Dissolve crystal of carbolic acid in chemically pure sulphuric acid to satuation and add hot distilled water until diluted for use as may be desired.

**Sodium Dioxid.** A solution of this agent will disintegrate impacted tooth pulp which may cling to your nerve broach or canal instruments in almost no time and leave the instruments as clean as a whistle.—Dr. Hatch, in *Cosmos.*

**Pyogenic Membrane.** Histologically there is no such thing as a pyogenic membrane. It is simply granulation tissue, an endeavor to form new structure. It fails to heal or form cicatricial tissue because of its environments.—Dr. Gilmer, in *Reviiw.*

**Dessicating Root Canals.** Dry cavities and root-canals with a hot air syringe or chip blower heated over an alcohol flame, and get the benefit of the formaldehyde gas generated by the decomposition of the alcohol for disinfectant purposes.—H. Almon, in *Review.*

**Temporary Dressing** Absorbent cotton saturated with cement mixed to the consistency of cream makes a good temporary dressing. It will last for months. If wanted for a short time, fill part of the cavity with dry cotton or medicated dressing and finish with the cotton and cement, as it will be more readily removed.—R. E. Sparks, in *Review*.

**New Dental Colleges.** Crighton University of Omaha, Neb., has organized a dental department with Dr. C. O. Metzler, as Dean. The State Dental College of Texas was incorporated February 28th, with $30.000.00 capital stock. Its home is to be in Galveston.

**Moustache Holder.** A strip of rubber dam two inches wide stretched over the mustache and tied back of the head will be a great convenience in working in the mouth of a person whose mustache obstructs the view especially of the lower back teeth. It can also be used to retract the lip and hold the napkin or cotton roll so as to expose the upper front teeth.—O. Martin, in *Review*.

**Drilling Sore Teeth.** When necessary to drill into a pulp chamber having an acutely-inflamed peridental membrane, take two pieces of soft modeling compound and adapt them to the lingual and buccal surfaces of three or four adjacent teeth. When cold they will serve as splints to hold the teeth firmly, and prevent the irritation incident to the drilling of the tooth.—Dr. Gilmer, in *Review*.

**Ligature Wire.** The use of wire ligature in approximating fractures of the jaw to get successful results implies that the ligature should neither stretch of break. German silver wire is preferable to brass or silver. No. 22 or 24 gauge wire should be used. It should be annealed by inserting in plaster of Paris and pumice and heating for fifteen minutes with a blow-pipe.—Arthur Black, in *Cosmos*.

**Water Sterilization.** Water may be sterilized by the addition of one part of silver fluoride to every five hundred thousand parts of water. A slight turbidity takes place on the addition of the fluoride, which clears up after twenty-four hours. The amount of silver used is so slight as to be of no harm. The amount of contamination in the water and character will of course influence the amount of the fluoride used.—*Chemist and Druggist.*

**Precipitating Gold from Other Metals.** Dissolve the metals and make the solution thoroughly acid with either nitric or hydrochloric acid. Then add from ten to one hundred cubic centimeters of commercial formalin, according to quatity of solution. The action is hastened by gentle heat. Pure gold is precipitated from the solution in crystalline form, though it contains mercury, zinc, lead, tin and other metals.—F. J. McInnes, in *British Journal.*

**Button Your Cement.** The *Office and Laboratory* suggests that it is a good plan to make a test button of each color in a new package of cement so that you may know exactly what color to select for a given case. Polish the button as you would a filling and just before testing for the color wet the cement button, as it will then more nearly resemble the tooth conditions. Be careful to have the mixing slab clean and use a non-corrosive spatula, as these may greatly modify the color.

**Dry Treatment of Burns.** Sattler (*Wien. med. Presse*), reports one hundred cases of burns treated with xeroform, which is freely dusted over the inflamed surface and then covered with powder and dressings. These are left undisturbed for six days, and then redressed in a bath. The treatment commends itself because (1) it alleviates pain and distress rapidly; (2) the wounds clean rapidly; (3) the raw surfaces are quickly covered with healthy granulations and the epidermis rapidly closes in.

**Method of Cleaning and Polishing German Silver.** German silver is very often used in dentistry, but is rather hard to swage and polish. While working this metal I anneal it by heating to a red heat and plunging in a in solution of oxalic acid. This cleans and softens the metal better than any method I know of. When the appliance is ready to polish I employ Burnishine or Solarine instead of pumice or whiting. I accidently discovered this method and find it effective.—C. J. Hadley, in *Review*.

**Johnson's Lip Pinned.** The editor of the *Dental Review* recently visited his old country, and took in the Ontario Dental Society meeting. It happened that a banquet was served on the evening of Johnson's birthday. As an expression of their great pleasure that he had ever been born, and particularly that he had selected Canada for his birth place, the members of the society presented him a handsome scarf pin suitably set with gems. It was too much for the modest editor, and spoiled one of his most beautifully canned orations.

**Polishing Crowns.** Fit a pine stick loosely into a gold shell crown, then coat the fitted end of the stick with hot gum shellac. Fill the interior of the crown with soft plaster to which salt has been added to make it set quickly, and insert the end of the stick with the soft shellac. Cool, and it is ready for the buff wheels. It will hold securely and when you are through heat the crown and it will easily come off. The hard plaster shell will easily crack away, leaving the interior of the crown bright and clean.—*Office and Laboratory*.

**Keeping Instruments Sterile.** I have found nothing that has seemed to be so useful for this purpose as formaldehyde. I am experimenting with the full strength formaline and solutions made from the tablets. I am not sure that the tablet solutions are strong enough. I have not found formaline so detrimental to instruments as some operators. They can not remain long in a concentrated solution, but no harm will come from their confinement in a case where the vapor will come in contact with them.—Dr. Rhein, in *Cosmos.*

**Trichloracetic Acid.** I wish to speak a good word for trichloracetic acid in dental practice, because I believe it has a wider range of application than any other single preparation. First, it is most excellent in the treatment of pyorrhea, arresting the accumulation of pus in very short order. In the treatment of putrescent pulp-canals it acts like a charm; carefully applied to spongy gums it gives better results than anything else; in pericementitis arising from calcic deposits it is excellent. It is both escharotic and astringent and it destroys abnormal surface tissue and purifies the same in a few moments after being applied.—H. C. McK., *Brief.*

**Report of Ohio State Examining Board.** The board issued in 1904, 218 certificates to practice. 175 of these were issued to graduates of Ohio colleges who are exempt from examination up to the June, 1905, examination; 19 were granted their certificates on examination, and 17 were registered under exemption clauses of the law. Seven duplicate certificates were issued. Six cases of prosecution of illegal practitioners were successfully completed. One certificate fraudulently obtained six years ago was recovered on demand and the man who obtained it has left the State. The income for the year was $3.325.56, and the disbursements $2,251.23.—*Summary.*

**Iodine and Chinosol Contra-indicated.** Dr. J. P. Buckley, in a reported discussion in the April *Review*, says, there are so many drugs that can be used in canal disinfection that we are hardly justified in using such straining agents as iodine, chinosol, etc. These agents when applied with steel probes will most likely discolor the tooth. Some operators may be so skilful that they can introduce such drugs to the apical end of the root without endangering the coronal portion of the tooth, but most of us can't do it. Use remedies that will not decompose color pigments, and seal them into the teeth with cements for a few days. Be careful not to force any decomposed pulp tissue or infection through the apical foramen.

**Bridge Repair.** It occasionally happens that a porcelain facing breaks away from its backing on a molar or bicuspid leaving a backing too thick to drill holes and replace the facing by riveting the pins or using nuts on the threaded pins. Select a suitable facing and turn the ends of the pins together so as to form a loop and solder. Cut a slot in the gold backing of the bridge iarge enough to admit the loop and let the facing go to place. Measure the extent the loop will penetrate the gold backing by placing the facing against the backing with the loop extending over the occlusal surface of the dummy. Drill a hole through the gold cusp perpendicular extending into the slot so that a pin passed through this hole will engage the loop of the facing. Fit a gold wire snugly into this hole. Then cement the facing to place and while the cement is yet soft drive the gold wire into the hole until it engages the loop.—R. E. Sparks, in *Review*.

**Porcelain-faced Incisor Crown.** Make an all gold shell crown, either seamless or soldered, and cut out the labial face to accommodate a porcelain tooth slightly smaller than the tooth required. Grind the lingual surface of the porcelain until the pins have been entirely removed and only a veneer of porcelain remains. Back this porcelain veneer with 30-gauge pure gold, covering the entire labial surface and allowing the edge of the backing to extend over the border just enough to clamp it to the facing. Burnish it closely to place after it has been accurately trimmed to size, and then fit it into the space cut out of the labial face of the gold crown as accurately as possible. Invest and solder from the interior with 22-karat solder. With suitable sandpaper and cuttlefish disks dress down the seams where the margins are united, running the disk toward the porcelain so as to burnish the gold over its margins. This makes an artistic and durable jacket crown, and is not difficult to make.—T. O. McCallie, in April *Cosmos*.

**Mummy Paste.**

R. Arseneous Acid.............grs. v.
Tannic Acid............ " x.
Acetate of Morphine...... " v.
Mix and triturate.

Sig. Moisten a very small ball of cotton with carbolic acid and roll it in this powder and apply to the exposed pulp. Seal it with cement or gutta-percha for one week. At the end of this time the entire pulp can be removed with a barbed broach dead and dried. I once made a dressing of this to a tooth which I did not see again for a year, and when I opened into the canal found it sweet and clean. I made a dressing of this kind for a lady last summer and she won't let me remove it as she says it suits her well enough. When I find a root-canal that I can't remove the entire pulp from, I make a dressing of this paste and fill over it permanently with no bad results. I have made this treatment hundreds of times in the last fifty years and have yet to learn of a bad outcome.—Mossback, in *Texas Journal*.

**Hones for Dental Instruments.** The hone should not be less than six to eight inches long, two inches wide and from one to one and a half inches thick. A medium grit is the best for general use. The Turkish stone is best but very hard to get. The Arkansas stone is good but rather hard. The Washitaw stone is next to the Turkish hone. The manufactured stone called the "Franz Swatey," is good for fine edged instruments.

Oil stones improve with age, if kept clean and filled with oil. If not kept clean its surface accumulates particles of steel and the oil hardens in its surface destroying its grinding properties. A saturated solution of caustic potash in water is a good cleansing agent. Continued use of kerosene oil hardens the surface. Sperm oil is the best lubricant. If the surface becomes grooved or uneven, it may be dressed by rubbing it on a sheet of sandpaper which is fastened to the bench. Use coarse paper at first and fine paper at the end. Case the stone in a box so as to keep extraneous material from accumulating on its surface. Put in a good light near the operating table, and make it convenient to sharpen instruments without trouble or delay.—B. Bannister, in *Summary*.

**Aseptic Canal Filling.** The canal should be first cleansed of all extraneous and organic material by aseptic instrumentation. I then apply sodium-potassium, which will thoroughly disintegrate any organic tissue which may cling to the canal walls. The canal is then thoroughly washed out with a one to one thousand solution of bichloride of mercury slightly acidulated with tartaric acid. When the canal is dried out it will be found that owing to the acid reaction on alkaline sodium and potassium some of the mercuric chloride will have been deposited on the walls of the canal, where it remains a continual antiseptic reagent of the best sort. Care must now be taken to introduce no septic influence in filling the canal. The filling material should be sterilized as well as the instruments used. The filling material can be sterilized in formaline and the instruments when cleaned should be dipped in alcohol and passed through the flame of a lamp. This will completely sterilize the instrument and prevent carrying infection into the canal. Of course each step must be carefully executed with clean hands and with every possible precaution, especially while placing the filling material in the apical opening.—Dr. Rhein, in *Cosmos*.

**Napkin and Cotton Roll Combination.** The *Office and Laboratory* for March illustrates a method of using these absorbents advantageously. Two sections of roll are cut about an inch in length and placed one in each of two corners of a small antiseptic napkin which is then made into a roll. The center is less bulky and by twisting this part of the roll is much reduced in size, so that when the ends which contain the cotton roll are placed on the lingual and labial surfaces, the twisted or reduced part of the roll which has no cotton in it passes over the teeth and so does not obstruct the operator's view of the operation. It can be held in place by the fingers or a clamp as may be desirable. We would suggest that the cotton roll be cut diagonally instead of at right angles, and that the diagonal ends be placed toward the center. This will avoid the square shoulder that may be in the way at the point of the operattion. We would also suggest that the farther ends of the cotton roll be placed nearly an inch away from the corner of the napkin, so that the sides of the napkin may be turned up over the ends of the cotton before rolling. This will prevent the cotton from slipping out of the napkin and prevent the raveling of the napkin. These threads often get in the way and are a source of annoyance. Where a long roll is required, place the cotton on the diagonals of the napkin.

**Fusing Porcelain.** Mr. R. Brewster, in the April *Cosmos*, gives the following suggestion as to fusing porcelain which are well worth considering. Porcelain that is insufficiently fused to develop its full color is not as strong as it should be. At 1700 degrees, which is about red heat of the electric furnace, the porcelain is white in color and just hard enough to hold together for filing to definite form. At 1800 degrees, or bright red heat, it can still be filed, but shows a little color. At 1900 degrees it is of a brownish color and too hard to file. At 2016 degrees the fusing point of gold, it assumes a yellow color and the surface is much smoother than before. At about 2100 degrees the color comes out bright and the surface becomes smooth and glossy. Carrying the heat still higher more completely fuses the porcelain and produces a glass surface, and threatens the form of the porcelain. The strength of porcelain is not weakened by excessive fusing. Porous porcelain is weaker than solid or overfused porcelain. Porosity is caused by inserting the wet porcelain paste into the furnace before it is thoroughly dried out. The heat of the furnace forms a crust on the surface which prevents the escape of the steam generated in the interior of the mass, which causes the grains of porcelain to separate leaving spaces which are not obliterated when the porcelain fuses. Don't lay the matrix filled with the wet paste on a hot slab as it will lift the paste out of the matrix and leave a space or bubble. Use a clean, bright spatula to mix the porcelain paste and work it as dry as possible, and jar to place. A soiled spatula will certainly effect the color of an inlay. Dry the porcelain as thoroughly as possible before putting it into the muffle, then let it heat up to the furnace heat before increasing the heat. When the heat of the inlay has reached that of the furnace, the heat may then be increased as rapidly as desired without damage to the material. Each furnace and its current must be studied by the operator. When it is once determined what time is required for fusing, counting from the fusing point of pure gold, it will be easy to secure uniform and accurate results. Time should never be counted from the cold furnace. After the porcelain is fused it should not be withdrawn from the muffle too soon. The heat should be allowed to go down so that the molecules of the porcelain become permanently adjusted, before the piece is removed from the furnace. The gasoline furnaces are well adapted to baking small pieces of porcelain and can be worked accurately by the gold test and time.

